# 3D Magnetic Field Reconstruction Methodology Based on a Scanning Magnetoresistive Probe

**DOI:** 10.3390/s18072049

**Published:** 2018-06-27

**Authors:** Filipe Richheimer, Margaret Costa, Diana C. Leitao, João Gaspar, Susana Cardoso, Paulo P. Freitas

**Affiliations:** 1 INESC-Microsistemas e Nanotecnologias (INESC-MN), 1000-029 Lisboa, Portugal; dleitao@inesc-mn.pt (D.C.L.); scardoso@inesc-mn.pt (S.C.); 2 Physics Department, Instituto Superior Tecnico, Universidade de Lisboa, 1049-001 Lisboa, Portugal; paulo.freitas@inl.int; 3 International Iberian Nanotecnology Laboratory (INL), 4715-330 Braga, Portugal; margaret.costa@inl.int (M.C.); joao.gaspar@inl.int (J.G.)

**Keywords:** magnetic field reconstruction, scanning magnetoresistance microscopy, giant magnetoresistance, spin valve, 3D magnetic field

## Abstract

The present work provides a detailed description on quantitative 3D magnetic field reconstruction using a scanning magnetoresistance microscopy setup incorporating a 19.5 μm × 2.5 μm magnetoresistive sensor. Therefore, making use of a rotation stage, 11 nm thick ferromagnetic CoFe elements with 20 μm × 5 μm planar size were measured along different sensor axes and converted into cartesian coordinate magnetic field components by use of the analytical coordinate transform equations. The reconstruction steps were followed and validated by numerical simulations based on a field averaging model caused by a non-negligible sensor volume. Detailed in-plane magnetic component reconstruction with ability to reconstruct sub-micrometer features is achieved. A discussion on the limiting factors for optimal resolution is presented.

## 1. Introduction

The imaging of magnetic stray fields in the micro- and nanoscale is required in a variety of fields and applications, ranging from the detection of nanoparticle tags on biomaterials [1,2,3], magnetic domains and domain wall propagation [4], high density magnetic storage imaging [5,6] or imaging stray fields produced by electrical currents [7,8]. Likewise, there is a range of applications that require measurements that go further than the conventional qualitative and unaxial magnetic field intensity detection. Examples are the accurate determination of arbitrarily oriented magnetic moments of submicron features for novel sensor applications [9] or for further postprocessing of the acquired images, as for example by application of the inverse problem for fault detection in 3D stacked electronics [10].

To study the aforementioned, a broad range of techniques are available, depending on the requirements of sensitivity, lateral resolution or measurement conditions. While scanning quantum interference device (SQUID) based microscopy [11,12,13] allows to achieve the highest magnetic flux detection sensitivities, measurements are based on superconductors in a Josephson junction configuration, meaning that operating temperatures are limited by the critical temperature of the respective superconductor material. In addition, although resolution in the tens and hundreds of nm have been achieved by improving sensor dimensions [14], it still remains a challenge to consistently measure the sub-micrometer scale using SQUID. Lateral resolution is also the limiting factor of scanning Kerr microscopy [15], as the polarization rotation of light is used as the magnetic field measurement principle, imposing diffraction limited resolution.

Furthermore, another class of micro and nanoscale sensors are based on the integration of magnetic field sensing into scanning probe microscopy (SPM) setups. SPM is an established technique in nanocharacterization, initially focused on high resolution measurements of surface topography first through scanning tunnelling microscopy [16] and a few years later with atomic force microscopy (AFM) [17], allowing sub-nanometer vertical resolution in ambient conditions. In addition to precise surface profile measurements, SPM offers a straightforward measurement of physical quantities simultaneously depending on the tip properties, giving rise to electrical [18,19,20], chemical [21,22,23] and magnetic modes [24], amongst many others.

Two different strategies were pursued for this purpose. On the one hand, by covering conventional SPM probes with a magnetic coating, known as magnetic force microscopy (MFM) [24,25], or by integrating dedicated magnetic field sensors into the vicinity of the SPM probe tip, for example scanning hall probe microscopy (SHPM) [26,27] or scanning magnetoresistance microscopy (SMRM) [28]. Out of these, MFM is the most frequently used. Firstly, this is due to the fact that MFM sensors, which can be conventional AFM probes with tips coated by a ferromagnetic material, are broadly available commercially. Moreover, it allows high resolution in the tens of nanometer range [29], which is lower than the other two magnetic SPM modes mentioned above. Nonetheless, since MFM measurements are based on the detection of the force interaction between the magnetic tip and sample, it is not straightforward to quantify the measured signal into a magnetic field intensity. Another challenge in MFM is the acting of the magnetic tip on the sample caused by the short distance [28].

The ability of quantitatively measuring magnetic field intensities in ambient conditions without requiring sample preparation is the significant advantage of SHPM and SMRM. Both techniques rely on the integration of different magnetic sensors, hall and magnetoresistive sensors. Significant advantages of the use of magnetoresistive sensors are sensitivity at ambient conditions avoiding the need of amplification [1,30] coupled to the ability to measure fields in all directions with a good degree of directionality (i.e, distinguish *x*, *y* or *z* fields) in a large variety of geometries [31]. Nanoscale resolutions on magnetic nanomaterials have already been achieved by attaching a commercial read and write head of conventional hard disk drives on a raster scanning setup [32]. Hall sensors integrated into a quartz crystal fork probe showed quantitative magnetic field imaging simultaneous to topography measurements on hard disk specimen with microscale resolution [33]. Nonetheless, the use of orientation sensitivity of these types of sensors in SPM applications appears to be unexplored so far.

In the present work, we demonstrate the potentiality of quantified 3D magnetic stray field reconstruction using a magnetoresistive spin valve (SV) sensor [34,35,36] integrated in an AFM probe. For this purpose, a measurement procedure is introduced which will be validated by numerical simulations at each step. This approach offers the potential to fully describe the magnetic flux intensity vector close to the sample surface of submicrometer features, allowing quantitative magnetic imaging at ambient conditions simultaneous with precise topography measurements.

## 2. Materials and Methods

### 2.1. Integrated Sensor

The SV sensor consists of the stack 2Ta/$3\;{\mathrm{Ni}}_{80}{\mathrm{Fe}}_{20}$/$8.5\;{\mathrm{Mn}}_{78}{\mathrm{Ir}}_{22}$/$2.3\;{\mathrm{Co}}_{80}{\mathrm{Fe}}_{20}$/0.8Ru/$2.3\;{\mathrm{Co}}_{80}{\mathrm{Fe}}_{20}$/2.7Cu/$2.3\;{\mathrm{Co}}_{80}{\mathrm{Fe}}_{20}$/$3.6\;{\mathrm{Ni}}_{80}{\mathrm{Fe}}_{20}$/5Ta (thickness in nm, film composition in atomic %) deposited on a Si/Al_2_O_3_ 100 nm substrate. Details on the film deposition can be found in [37]. The sensor was patterned with direct write laser lithography and ion beam milling [38] using a 105 mA Argon beam (≈340 μA/cm${}^{2}$) at 70° from the substrate surface. The sensor contact was done with a 2-contact configuration, using a 300 Al_98.5_Si_1.0_Cu_0.5_/15 Ti_10_W_90_(N) stack deposited by magnetron sputtering and defined by liftoff. Its field response was studied by placing the SV inside a uniform magnetic field, allowing to read out an electrical resistance *R* = 150Ω and a sensitivity of the transfer curve in the linear region (±2 mT) $\frac{dV}{dH}=856\frac{\mu V}{\mathrm{mT}}\;\left(\frac{dR}{dH}=856\frac{m\Omega }{\mathrm{mT}}\right)$, with a magnetoresistance ratio of MR=5.49%.

**Figure 1:**
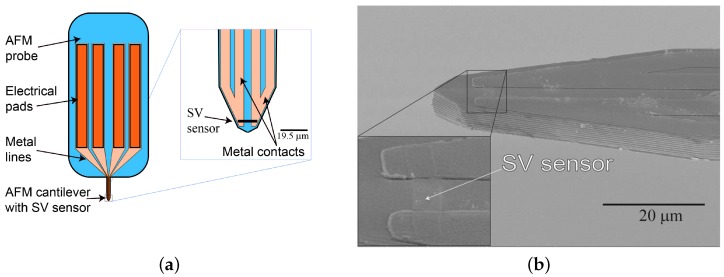
Integration of the sensor in the AFM probe. Schematic of the probe chip (**a**), scanning electron microscopy image of the cantilever tip with the integrated SV sensor (**b**).

Embedded into the front end of the AFM probe cantilever, the SV had in-plane dimensions of 19.5 μm × 2.5 μm, with the sensor long axis being perpendicular to the cantilever axis [39]. The sensor response was linearized by using shape anisotropy with a perpendicular reference layer orientation. As a result, the sensitive axis of the SV was oriented parallel to the short axis of the cantilever. The fabrication process of the SMRM probe is described in detail for the first generation in Ref. [40]. After the SV sensor deposition and patterning, a 100 nm thick Al_2_O_3_ is sputtered onto the SV sensor as a passivation layer. This passivation layer protects the patterned SV sensor from the hydrofluoric acid (HF) release after machining the AFM cantilever by low temperature deep reactive ion etching (DRIE). By wire bonding the metal tracks of the AFM probe onto a custom flexprint PCB, electrical contact was ensured. Figure 1 shows a schematic of the chip mounted as it will be mounted onto the AFM head (Figure 1a) and a scanning electron microscopy image (Figure 1b) of the embedded SV sensor contacted by two metal tracks.

This second generation of integrated magnetic sensors differs from the first in design aspects. It was identified that, for application purposes, a single sensor with a magnetic field sensitive axis colinear to the cantilever axis would facilitate the deconvolution of the magnetic field intensity into its Cartesian components. Furthermore, the SV was integrated into a significantly softer cantilever, with the mechanical properties being a resonance frequency of $f_{\mathrm{res}}=31.1$ kHz and a spring constant *k* = 3.36 N/m.

### 2.2. Scanning System and Electronics

The scanning system used in this work was a commercial, however customized, AFM system developed by Nanosurf (Nanosurf AG, Liestal, Switzerland), which accommodates electronics capable of simultaneously processing topography data and MR sensor signals. The AFM head has a two-dimensional scan range of 100 μm on each axis with a drive resolution of 1.53 nm on the *x*–*y* plane and approximately 10 times smaller along the *z*-axis with 0.15 nm. A translation stage is attached to the head with a travel range of 13 mm in each spatial direction, having a re-positioning precision and straight line accuracy both below 10 μm. When the cantilever is mounted on the AFM head, it has an angular elevation of 10° towards the *x*–*y* sample plane.

**Figure 2:**
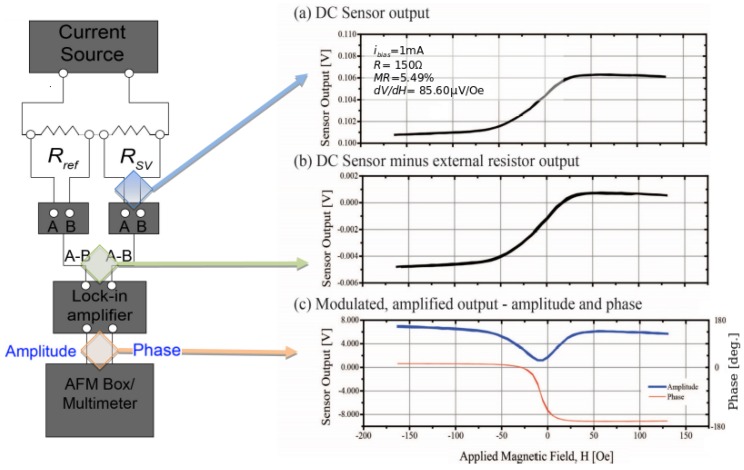
Effects of the electronic setup on the magnetoresistive signal under uniform magnetic field. **Left:** electrical circuit processing the signal measured by the SV sensor. **Right:** (**a**) magnetoresistive transfer curve at 1 mA bias current; (**b**) DC offset removal by tunable reference resistor before feeding into lock-in amplifier; (**c**) signal demodulated by the lock-in amplifier set at bias current frequency.

The electronic setup consists of a closed circuit between an AC current source connected to a variable resistance and the sensor head in series. A visual representation of the circuit is presented in Figure 2.

At each relevant circuit node, the profile of the sensor transfer curve is presented. The transfer curve was generated by sweeping a well-known magnetic field generated by a Helmholtz coil. The first node, corresponding to the blue arrow, shows the voltage output coming directly out of the sensor fed with a 1 mA AC bias current, generated by a Keithley 2400 SourceMeter (Keithley Instruments, Solon, OH, USA), when exposing the sensor to the point by point magnetic field sweep which can be considered as static (DC) compared to the bias frequency of the sensor.

The arrow pointing towards the transfer curve (Figure 2b) after correcting the voltage offset caused by the internal resistance of the sensor. This is achieved by using a nominal resistance connected in series, with both signals connecting to their individual Stanford Research Systems SR550 FET-input preamplifier (Stanford Research Systems, Sunnyvale, CA, USA) to avoid signal-to-noise ratio degradation of the sensor signal. The signal is zeroed by feeding the differential output of each preamplifier into the A-B input of a Stanford Research Systems SR510 lock-in amplifier.

Subsequently, the AC signal is decomposed into amplitude and phase signals by comparing the sensor output signal with its AC bias current. The transfer curves after lock-in amplification are highlighted by the arrow directing towards graph (Figure 2c). The phase signal shows a very smooth transition from a saturated in-phase behaviour to a saturated out of phase regime. This was not expected, as in the lock-in decomposition the phase encodes the orientation of the applied magnetic field towards the SV field sensitive axis. We expected a sharp transition around the zero field (H=0). The measurements of the transfer curves in Figure 2 were acquired using an AC sensor bias current at 50 kHz. Frequency dependent distortion caused by reference resistor and sensor components were considered to be responsible for the artifact in the phase signal, most likely due to inductive impedance contributions. These parasitic effects motivated a more detailed analysis to ensure an accurate transfer curve for calibration.

It was found that significantly reducing the bias current AC frequency eliminated the artefact in the transfer curve. Table 1 summarizes the parameters that were identified as appropriate taking into account the magnetic properties of the samples used in this study, considering map scans of 100 μm × 100 μm. Here, the integration time refers to the adjustable time constant of the low-pass RC filter, sensitivity refers to the gain and time/line quantifies the scanning speed of the AFM.

### 2.3. Ferromagnetic Samples

In order to be able to validate the measurements and field component reconstruction with simulations, a well controlled sample of ferromagnetic test structures was used. These patterned microstructures are periodic cuboidal arrays of thin film permanent magnets of different sizes and form factors. In more detail, the evaluated magnetic structures consist on Si (substrate)/100 Al_2_O_3_/5 Ta/11 Co_90_Fe_10_/5 Ta/5 Ru/15 TiWN/25 SiO_2_ (thickness in nm, film composition in atomic %). The top SiO_2_ layer was used to protect against corrosion and mechanical erosion if in contact with the probe. Different form factors were available on the full sample structure, and this work will focus on the 20 μm × 5 μm dimensions as they were approximately the lateral size of the sensor. To prove the ability to reconstruct magnets with submicrometer spatial dimensions, we will also show an example for 5 μm × 1 μm sized features. All magnetic features had permanent magnetization along the *x*-axis of the laboratory coordinate system, which in the case of the 20 μm×5 μm structures corresponds to an orientation along the long axis.

### 2.4. Methods

**Figure 3:**
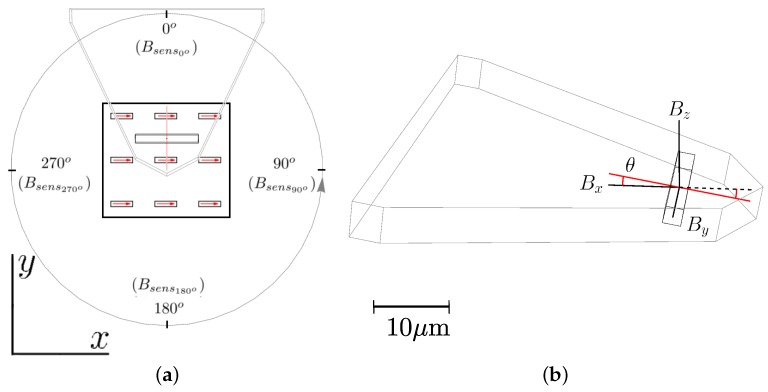
(**a**) illustration in the top view of the stage rotation for field sensing along different axes. Grey arrows define stage rotation direction, and red arrows show magnetization orientation of the patterned features at the laboratory frame indicated by the coordinate axes; (**b**) polar angle θ projecting the cantilever tilt on the SV sensitive axis.

Profiting from a controlled rotation of the measurement stage, the magnetic field reconstruction was based on measuring the same sample along different sensitive axes of the SV sensor. The cantilever orientations at which the sample was acquired and the notations for the magnetic field intensities sensed at each measured angle are described in Figure 3a. Figure 3b shows the vertical angle of the sensitive SV axis caused by the tilt of the AFM cantilever towards the sample plane.

The sensitive axis of the SV sensor is along its short axis, which gives two possible orientations, signalized by the two red collinear lines leaving the centre of the sensor in opposite directions in both figures. The correct SV orientation will be identified later in this work by comparing the measured image with simulations of both possible sensitive orientation configurations.

At this point, however, since the sensitive orientation was not known, both possible sets of equations at each measurement position are presented. These represent the coordinate system transformations of the magnetic field intensity from the sensor frame into coordinates of the laboratory.

We distinguish the two possible sensing orientations and assign the index (+) to the direction from the center of the sensor pointing inside the AFM cantilever, and (−) to the orientation from the center of the sensor to the tip and outwards of the cantilever. Accordingly, the set of equations that relate the sensed fields along the (+) direction with the magnetic flux density of the laboratory will be given by the decomposition of the sensing direction into the contribution of the laboratory frame components, resulting in the following set of equations:

(1) $$\begin{array}{c} \left\{\begin{array}{cc} B_{{\mathrm{sens}}_{{0{}^{\circ}}_{+}}} & =\;\;\;\;\;B_{y}\times \cos\left(10{}^{\circ}\right)+B_{z}\times \sin\left(10{}^{\circ}\right)\\ B_{{\mathrm{sens}}_{{90{}^{\circ}}_{+}}} & =\;\;\;\;\;B_{x}\times \cos\left(10{}^{\circ}\right)+B_{z}\times \sin\left(10{}^{\circ}\right)\\ B_{{\mathrm{sens}}_{{180{}^{\circ}}_{+}}} & =-B_{y}\times \cos\left(10{}^{\circ}\right)+B_{z}\times \sin\left(10{}^{\circ}\right)\\ B_{{\mathrm{sens}}_{{270{}^{\circ}}_{+}}} & =-B_{x}\times \cos\left(10{}^{\circ}\right)+B_{z}\times \sin\left(10{}^{\circ}\right). \end{array}\right. \end{array}$$

Repeating the same process for the sensing direction pointing in the opposite direction, we obtain:

(2) $$\begin{array}{c} \left\{\begin{array}{cc} B_{{\mathrm{sens}}_{{0{}^{\circ}}_{-}}} & =-B_{y}\times \cos\left(10{}^{\circ}\right)-B_{z}\times \sin\left(10{}^{\circ}\right)\\ B_{{\mathrm{sens}}_{{90{}^{\circ}}_{-}}} & =-B_{x}\times \cos\left(10{}^{\circ}\right)-B_{z}\times \sin\left(10{}^{\circ}\right)\\ B_{{\mathrm{sens}}_{{180{}^{\circ}}_{-}}} & =\;\;\;\;\;B_{y}\times \cos\left(10{}^{\circ}\right)-B_{z}\times \sin\left(10{}^{\circ}\right)\\ B_{{\mathrm{sens}}_{{270{}^{\circ}}_{-}}} & =\;\;\;\;\;B_{x}\times \cos\left(10{}^{\circ}\right)-B_{z}\times \sin\left(10{}^{\circ}\right). \end{array}\right. \end{array}$$

## 3. Numerical Implementation

**Figure 4:**
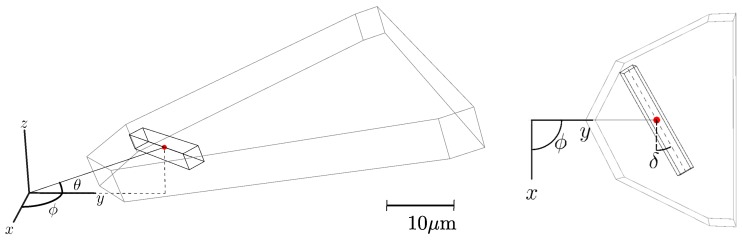
Angular degrees of freedom given in the computational implementation of the model.

The model is focused on replicating the response of a field sensor according to its physical properties during the measurement. Therefore, the initial concern was to provide a set of degrees of freedom that allowed to position the sensor correctly in space, as demonstrated in Figure 4. From the fabrication conditions, the offset of the central point of the sensor towards cantilever tip was *d* = 11.4 μm along the cantilever long axis, with the sensor long axis placed transverse to the long axis of the cantilever (δ=0). The tilt of the cantilever towards the sample plane is θ=10°. The polar angle ϕ is left free and will be the coordinate that determines the different measurement orientations necessary for the deconvolution of measured magnetic field intensities into the Cartesian components of the laboratory frame.

The model implies that the SV sensor will measure the average of the external magnetic stray field enclosed by its volume along the sensitive axis defined by the reference layer orientation,

(3) $$\begin{array}{c} B_{\mathrm{avg}}=\frac{1}{V}\int_{V}\;\left(\vec{B}\cdot \vec{n}\right)\;dV. \end{array}$$

In addition, the simulation of the external magnetic stray field produced by the microfabricated permanent magnet structures was achieved by implementing a magnetic charge model for permanent magnets [41] reduced to in-plane magnetizations.

## 4. Results and Discussion

**Figure 5:**
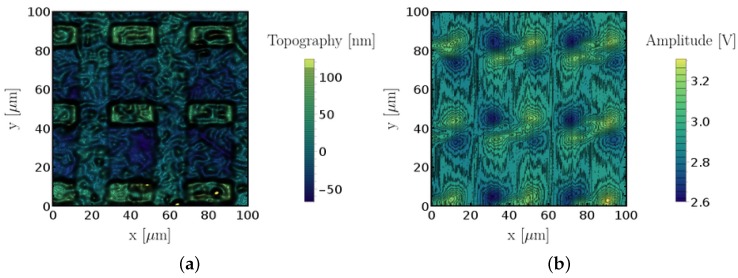
(**a**) topography and (**b**) amplified SV sensor output sensed at 0° for 20 μm × 5 μm magnetic features.

All measurements were performed in contact mode with topography and magnetic field intensity being recorded simultaneously. An example of measured topography and uncalibrated magnetic field intensity data is shown in Figure 5. Both images were linewise leveled to remove fluctuations of the set point in the AFM upon line changes. We can see the rectangular ferromagnetic features with a well defined height in the topography data and the corresponding magnetic field image patterns. The measurement noise of a calibrated magnetic image was estimated to lie between 5% and 10% of the measured signal amplitude by using a bilateral filter as explained in Appendix A. The vertical offset between the center of each figure in topography and amplified sensor signal is caused by the offset of the sensor towards the AFM tip.

### 4.1. Sensitive Axis Orientation

**Figure 6:**
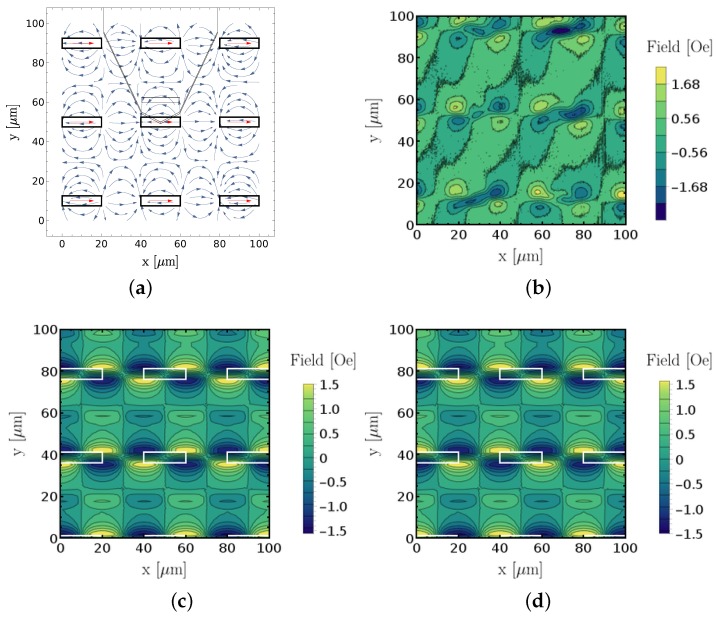
Comparison between measured and simulated magnetic intensities for sensitive axis determination. (**a**) graphical representation of measurement/simulation conditions; (**b**) measured and calibrated magnetic image; (**c**) simulated magnetic field intensity in (+) direction, white rectangles represent placement of permanent magnet features; (**d**) identical as (**c**) with simulated sensitive sensor axis along the (−) direction. Direction notation follows Equations (1) and (2).

As described in Section 2, the sensor sensitive axis is along the short dimension, leaving an ambiguity in its orientation towards the cantilever bulk or the tip. The strategy to identify the correct orientation was by comparing an experimental magnetic field intensity scan with numerical simulations of both possible sensitive axis orientations. Figure 6 shows the comparison between experiment and numerical results allowing us to conclude that the correct direction is (−) in our notation. This corresponds to the reference layer of the SV sensor pointing towards the AFM cantilever tip at zero field. In the experiment, minima are at top right and bottom left, while maxima are at top left and bottom right, which is correctly reproduced by simulations in Figure 6d. Hence, the sensed magnetic field intensities relate to the field components in the laboratory frame according to Equation (2). Alternatively, when features of well-known magnetization orientation are not available, the sensitive axis can also be determined under the application of an external magnetic field with a known direction.

### 4.2. 3D Magnetic Field Reconstruction

One major challenge to obtain 3D magnetic field reconstruction was to certify that the individual measurements with different sample rotations all had equal *x* and *y* coordinates. During measurement, if a certain region was chosen that did not coincide with the rotational center of the stage, the region would spatially move off position. This had to be compensated manually but could not be accurately verified. Furthermore, the offset between the SV sensor and the AFM tip meant that even if the spatial offset was matched in topography, the magnetic field images would still present a shift. In practice, this means that accurate phase matching can only be achieved during postprocessing of the data. In this experiment, the strategy pursued consisted of measuring larger areas (100 μm × 100 μm) covering several ferromagnetic features and cropping out a 40 μm×40 μm region with a magnetic pattern of a single feature at the center.

We identified that the simulated magnetic field intensities matched the experimental better when the magnetization of the individual features was simulated with an angle of 9° in counterclockwise direction from the *x*-axis. This value was reached by running a successive matching algorithm for different magnetization orientations. The magnetization values of the simulations were overlaid with the experimental maps and a global minimum of the sum of squared differences between magnetic intensities was found at that angle. Physically, this deviation from the horizontal axis can account for a deviation of the magnetic easy axis of the ferromagnets. This small angle correction will improve how both measured and simulated images correlate visually.

**Figure 7:**
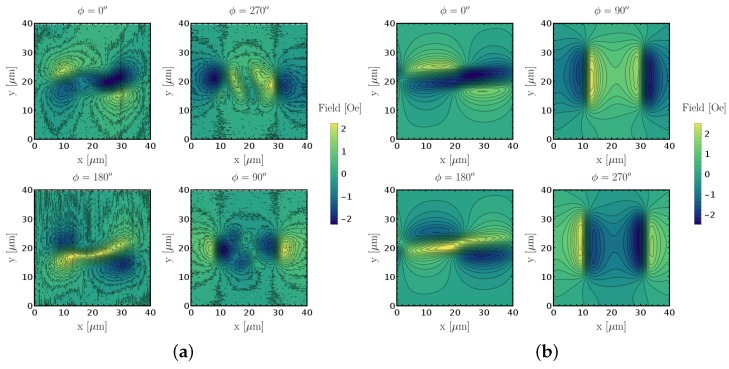
Single magnetic field intensity from single micropatterned 20μm×5μm feature. (**a**) measurement; (**b**) simulation.

Following these notes, the data used for magnetic field reconstruction are presented in Figure 7. Applying Equation (2), we can now deconvolve the respective $B_{{\mathrm{sens}}_{\phi_{-}}}$, where ϕ corresponds to the applied stage rotation, and thus solve the system of equations to determine $B_{x}$, $B_{y}$ and $B_{z}$ for each point in space, meaning that in total the linear system was solved $256^{2}$ = 65,536 times. This value corresponds to the total number pixels in each scan. To increase the computational speed of the reconstruction algorithm, the linear system solver was computed in parallel on the CPU.

**Figure 8:**
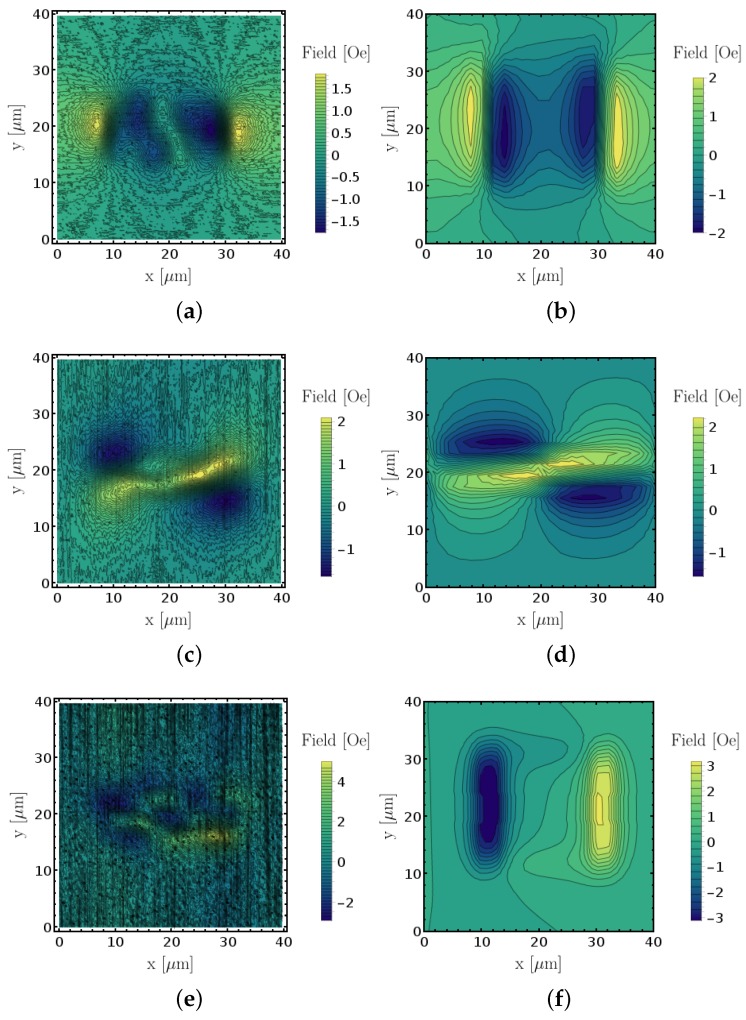
Comparison between measured and simulated magnetic field decompositions into cartesian coordinates of 20μm×5μm sized features. Measured and simulated $B_{x}$ (**a**,**b**), $B_{y}$ (**c**,**d**) and $B_{z}$ (**e**,**f**) components, respectively.

The experimental and simulated data was reconstructed individually and the results are presented in Figure 8. The results show good agreement between measurement and simulation for the in-plane magnetic field components $B_{x}$ and $B_{y}$, while the measured $B_{z}$ is not conclusive. This is related to the fact that the vertical out of plane component was only captured by a 10° component, hence the signal-to-noise ratio was considerably inferior compared to the other two axial components. The magnetic field intensities are also in close agreement in both cases. Most importantly, this means that the magnetic measurement with an SV sensor is well described as an average of the magnetic field inside the active area along the sensitive axis. It validates the model that can be used to predict improved sensor designs in future generations by identifying what sensor dimensions limit the for measurements resolution, at the micro- and nanoscale.

**Figure 9:**
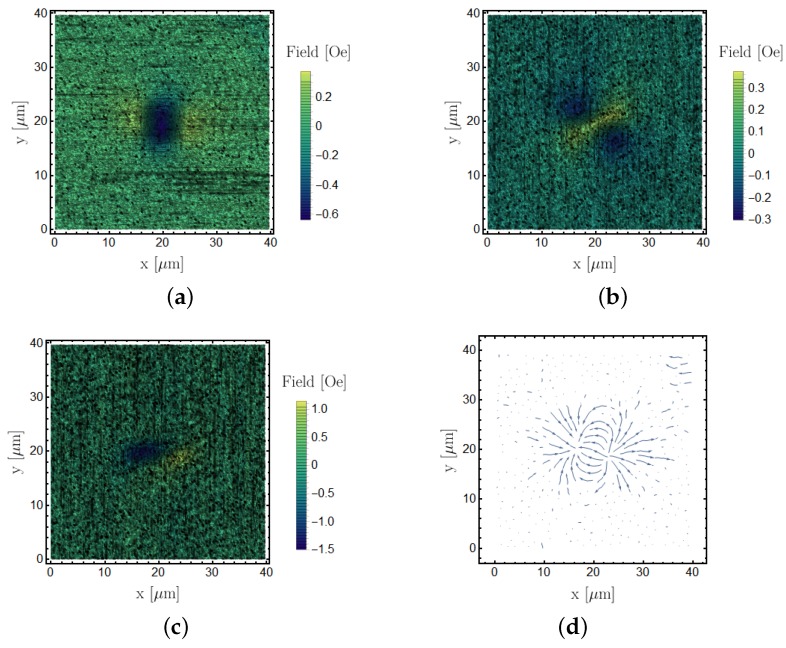
5μm×1μm CoFe feature magnetic field reconstruction. (**a**) $B_{x}$, (**b**) $B_{y}$, (**c**) $B_{z}$ and (**d**) is a representation of the magnetic flux of the stray field lines in the measurement plane by using the $B_{x}$ and $B_{y}$ components.

In Figure 9, we show a set of fully reconstructed magnetic images on a 5μm×1μm feature to demonstrate our capability of reconstructing sub-micron features with the current setup. Figure 9 shows an equivalent magnetic image pattern as the 20μm×5μm. We also combined the data of the $B_{x}$ and $B_{y}$ into a vectorial streamplot in Figure 9d, which shows the typical magnetic flux of a bar magnet.

Using the same sensing setup, we performed the reconstruction procedure for identical ferromagnetic CoFe features with smaller in-plane dimensions of 5μm×1μm. Figure 9 shows the resulting reconstructed laboratory frame components. The results were compatible with the larger 20μm×5μm feature, which were chosen for their stronger signal for representation purposes; however, it is possible to reconstruct magnetic domains below micrometer size with this method, as demonstrated in this section.

**Figure 10:**
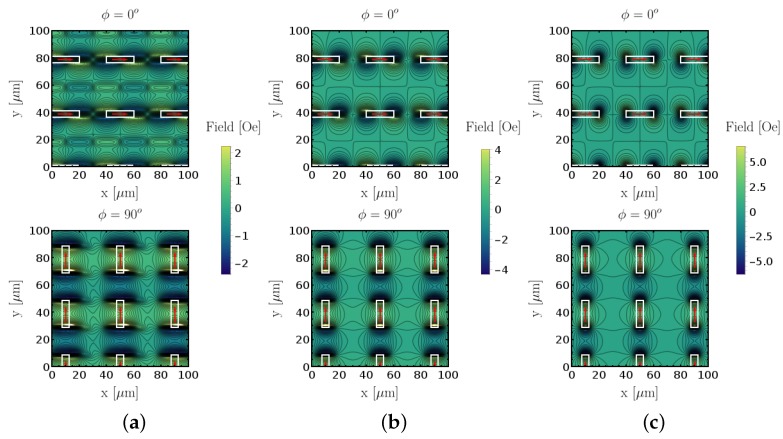
20μm×5μm feature sizes ($\phi_{\mathrm{mag}}$) simulated with different sensor base lengths and widths and constant height of 200 nm. (**a**) 19.5 μm × 2.5 μm sensor; (**b**) 9.75μm×1.25μm sensor; (**c**) point-like sensor.

To demonstrate the impact of sensor dimension in resolution, we compare the numerical simulations of a measurement on the 20μm×5μm sized patterned features using sensors with different planar dimensions. Therefore, we simulated images with the sensitive axis along two different orientations for each sensor size. The simulated sensor dimensions were identical to the physically available sensor (19.5 μm × 2.5 μm). For a comparison, the case was simulated when both planar axes were half the size (9.75μm×1.25μm). Figure 10 compares both cases to the point-like sensor, where zero volume was considered and the field response at each point was just the magnetic stray field intensity of the ferromagnetic features along the sensitive axis of the sensor. The averaging effect of the sensor volume is clearly visible in the measured magnetic field intensity. While the large sensor measures field intensities of less than half the magnitude of the ideal case, a quarter of the sensor volume only shows a total deviation of about 20%. In addition, the shape of the field patterns is more accurately reproduced by sensors with smaller size, which in the case of the larger SV sensor suffered from the appearance of artefacts in between the patterns created by the ferromagnetic features. These are not visible in the point-like case.

## 5. Conclusions

In this work, we demonstrated the use of SMRM devices for precise control of directional sensing of quantitative magnetic field intensities. Combining the measurements along three different orientations towards the sample, a reconstruction of the magnetic field intensity vector including all its three perpendicular components was possible—thus resulting in a complete magnetic characterization over an area defined by the scan size of 100 μm × 100 μm at constant height (≈3 μm) resulting from the vertical AFM tip to SV sensor distance. Due to the reduced angle between cantilever and sample plane (10°), the magnetic in-plane components showed significantly better signal-to-noise ratio than the out of plane component, which is in total contrast with competing magnetic scanning probe microscopy techniques like MFM, where mostly vertical field components are resolved.

The microfabricated sensor device on an AFM probe allowed for resolving patterned ferromagnetic features (11 nm thick magnetic CoFe layer) with approximately the same planar size as the active area of the sensor of 19.5 μm × 2.5 μm, both in the micrometre range. By introducing a field average model to mimic the volumetric sensor response on magnetic stray fields, each step of the magnetic field reconstruction procedure was validated by comparison between measured and simulated data. In addition, the model allowed for showing that magnetic resolution is limited by the sensor active area. The developed model also provides an estimation of the quantitative error on the measured magnetic field intensity caused by the field averaging effect.

The results of this work direct a route to micro- and nanoscale quantitative characterisation of magnetic samples, coupled to simultaneous topography measurements without significant crosstalk, with relevance to fields like nanometrology or spintronics. In order to drive these techniques towards lower resolutions and higher sensitivity, limiting factors such as the active area of the sensor and the vertical offset between AFM tip and SV sensor will need to be addressed.

## Figures and Tables

**Table 1:** Setup parameters used during magnetic imaging in this work.

Parameter	Value
Bias Current Frequency $\left[\mathrm{Hz}\right]$	1000
Integration Time $\left[\mathrm{ms}\right]$	10
Lock-In Sensitivity $\left[\mathrm{mV}\right]$	5
Time/Line $\left[s\right]$	1

**Figure A1:**
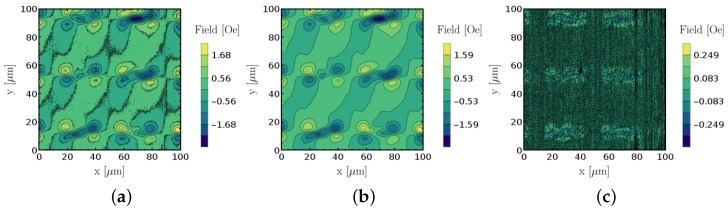
Bilateral filter for measurement noise estimation. (**a**) original 20μm×5μm features scan at ϕ=0; (**b**) 20μm×5μm features scan after applying bilateral filter; (**c**) subtraction of (**a**) from (**b**) as noise estimate.

## Abbreviations

The following abbreviations are used in this manuscript:

AFM	Atomic Force Microscopy
DRIE	Deep Reactive Ion Etching
GMR	Giant Magnetoresistance
MFM	Magnetic Force Microscopy
SHPM	Scanning Hall Probe Microscopy
SMRM	Scanning Magnetoresistance Microscopy
SPM	Scanning Probe Microscopy
SV	Spin Valve

## Appendix A. Noise Estimation by Filtering

To provide an estimation of noise, it is necessary to separate it from the signal. Our strategy relies on applying a bilateral filter from image processing, which has the properties to be a nonlinear edge preserving type of filter for signal smoothing. In Figure A1, we show the result of applying such a filter on the measured data (Figure A1a) of a 20μm×5μm features scan at ϕ=0. The result is shown in Figure A1b, where a significant reduction of noise is visible. Figure A1c is the subtraction of the original measurement scan from the filtered data at each pixel and is expected to be the noise component of the signal when considering that the filter was able to track the signal. However, we can see that, especially on the regions of magnetic field patterns of the features, artifacts from the original signal are visible. If we take into account that these artifacts also appear to contribute to the largest recorded amplitude in Figure A1c, our best estimation points to a measurement noise level of around 5–10%.
